# Time to recovery following cataract surgery and its predictors among patients undergoing surgery at two selected Public Hospitals in Hawassa, Sidama, Ethiopia

**DOI:** 10.1371/journal.pone.0313118

**Published:** 2024-11-04

**Authors:** Shilota Sarmiso, Dansamo Tediso, Temesgen Tafese, Taye Gari

**Affiliations:** 1 Department of Public Health, Hawassa College of Health Sciences, Hawassa, Ethiopia; 2 School of Public Health, College of Medicine and Health Science, Hawassa University Hawassa, Ethiopia; Dr. Agarwal’s Eye Hospital, INDIA

## Abstract

**Background:**

Cataract surgery is a commonly performed procedure aimed at restoring vision in individuals affected by cataracts. The duration of recovery following cataract surgery can vary among patients and is influenced by a multitude of factors. Therefore, the purpose of this study was to determine the time to recovery from cataracts and its predictors among patients treated with cataract surgery at two selected Public Hospitals in Hawassa, Sidama regional state, Ethiopia.

**Methods:**

Institution-based retrospective cohort study was conducted among 444 cataract patients treated with surgery from January 01, 2019, to December 30, 2021. A simple random sampling method was used to select two public hospitals. The data was collected using Kobo toolbox Version 4 and trained data collectors. STATA Version 16 was used for analysis. To estimate the recovery time and compare survival probability among variables Kaplan-Meir curve and Log-rank test were used. The cox-Proportional hazards model was used to identify significant predictors of time to recovery. The association was reported using the adjusted hazards ratio (AHR) with a 95% confidence interval (95%CI), and the significance level was set at a p-value of 0.05.

**Results:**

This study showed that 76.14% of cataract patients recovered from cataracts. The average time taken to recover from a cataract was 30 weeks (IQR = 15 to 48) 95%, CI, (26–33). Age 40–60 years (AHR = 2.04 CI; 1.12–3.82), urban dwellers (AHR = 1.48; 95% CI, 1.13–1.93), medium/high level of visual acuity (AHR = 1.49 CI; 1.14–1.94), secondary cataract (AHR = 1.56 CI; 1.11–2.18) and traumatic cataract (AHR = 1.82 CI; 1.32–2.52) were associated with time to recovery of cataract patients.

**Conclusions:**

According to this study, the time to recovery of cataract patients was slightly high. Cataract patients’ time to recovery was affected by age, residence, pre-operative visual acuity, presence of diabetes mellitus, and post-operative complications. To improve recovery time of cataract patients, treatment strategies must be prioritized.

## Background

Cataract is one of the leading causes of visual impairment worldwide, particularly among the elderly population [1]. In Africa, approximately 1% of the population was blind, with a higher prevalence estimated for Sub-Saharan Africa [2] including Ethiopia where the prevalence was 1.18%, with 50% of cases attributed to cataracts [3]. This highlights the significance of cataract surgery as a widely practiced and effective method for restoring vision [4].

The surgery involves the removal of the cloudy lens and the implantation of an intraocular lens, resulting in improved visual acuity and enhanced quality of life for patients [5]. Despite the evident benefits of cataract surgery, there are still important gaps in our understanding of the recovery process following the procedure. However, uncertainties persist regarding the recovery process post-surgery, impeding healthcare providers in effectively guiding and maximizing patient recoveries [6].

The amount of time needed to recover and return to everyday activities after cataract surgery varies greatly between individuals. Most patients experience gradual improvement over several weeks, but some may take longer [7]. According to a study done in Eastern Finland states the average recovery time of cataract surgery was 16 weeks and the same study done in Ethiopia states the average recovery time of cataract surgery was 23 weeks [8, 9].

While studies have investigated immediate postoperative outcomes such as visual acuity, patient satisfaction, and occurrence of complications, little attention has been given to the duration it takes for patients to achieve optimal visual recovery and regain functional independence [10]. The absence of comprehensive data on the time to recovery poses challenges for both patients and healthcare providers [11].

Another significant issue is the challenge of determining predictors associated with the recovery time from cataract surgery [6]. Various factors, including patient demographics, preoperative ocular characteristics, surgical techniques, and postoperative management, may influence the duration of recovery [7, 9, 12, 13]. However, there is a scarcity of studies that have systematically examined these predictors and their relationships with recovery time. Identifying these predictors is essential for optimizing patient care and developing personalized treatment plans. It would allow healthcare providers to identify patients who may be at a higher risk of delayed recovery or postoperative complications, facilitating early intervention and closer monitoring.

Moreover, the difficulties in identifying predictors of recovery time also hamper resource allocation and planning in healthcare facilities [7]. Without a comprehensive determining of the factors that influence recovery, it becomes challenging to estimate the resources and personnel needed to effectively manage the postoperative period. This may result in inefficient resource utilization, delayed patient care, and increased healthcare costs [11].

In light of these problems, there is a pressing need for research that addresses the time to recovery from cataract surgery and its predictors. It enables healthcare providers to set realistic expectations for patients, develop tailored postoperative care plans, and allocate resources more effectively identifying predictors of recovery time helps healthcare providers to identify high-risk patients and implement appropriate interventions [11].

Ultimately, addressing these gaps in identifying recovery time predictors will contribute to the advancement of cataract surgery practices and improve the overall quality of care for cataract patients. Therefore, this study aimed to determine the time to recovery from cataract and its predictors among patients treated with cataract surgery at two selected Public Hospitals in Hawassa, Sidama Ethiopia.

## Methods

### Study area

The study was conducted in a Hawassa city administration, which is the capital city of Sidama National Regional State. It is located 275 Km from Addis Ababa the capital city of Ethiopia. The city is subdivided into 8 sub-cities. The total population of Hawassa city in 2017 was 455,658 which are projected from the 2007 Census conducted by the Central Statistical Agency of Ethiopia [14]. Out of the four public hospitals and ten health centers, Adare General Hospital and Hawassa Comprehensive Specialized Hospital have been offering cataract surgery services for over three years, with established patient loads and high-quality data. Due to these factors, these two hospitals were chosen as the study area. Cataract surgery was provided by Ophthalmologists and Cataract surgeon. This eye care center provides all-inclusive clinical and community eye care health services for the region and serves as a major referral center that provides different specialty eye care services and training of eye care professionals such as Optometrists, Ophthalmologists, and ophthalmic nurses.

### Study design, period, and population

A facility-based retrospective cohort study was conducted from January 01, 2019, to December 30, 2021, and with data extraction taking place from April 01 to 30, 2023. The source population was all cataract patients treated with eye cataract surgery in Hawassa City public hospitals. The study population was all eligible population who has undergone cataract surgery in the two hospitals from January 01, 2019, to December 30, 2021. Those with incomplete medical records (cards that missed to register at least the following data: date of admission, status of the cataract patients, and other major predictors) were excluded from this study.

#### Sample size determination and sampling technique

The sample size was estimated using the double population proportion formula in the log-rank test of STATA software version 16 by considering different significant variables from the previous study, and by considering the following assumptions; the level of significance was 5%, and the power was 80%, P1 = Proportion of recovery among exposed and P2 = Proportion of recovery among the non-exposed group [9]. The ratio of the population exposed to non-exposed was 1:1. Variables such as residence, visual acuity, type of cataract, and type of surgery were used to calculate the sample size. Finally, the variable type of surgery was selected for final sample size estimation which gave a sample size of 444.

Records of 444 cataract patients, which were collected through retrospective review from January 01, 2019, to December 30, 2021 at Hawassa comprehensive specialized hospital = 330 and Adare general hospital = 114. Patients’ records were chosen through simple random sampling from the ophthalmic operation room logbook in each hospital using their medical registration numbers, and then their charts were reviewed.

### Data collection procedures

A standardized data extraction checklist was developed and used to extract information from patient cards. It was prepared in English and contained four parts, socio-demographic characteristics of the patients, behavioral factors (substance use), and clinical/surgical factors (level of visual acuity, type of cataract, eyes to be operated, types of lenses, post-operative complication, type of anaesthesia used for surgery, local anaesthesia, general anaesthesia, time taken for cataract surgery, types of cataract surgery), and medical factors. Data was collected by six ophthalmic nurses. The data extraction was additionally supervised by two first-degree public health professionals.

### Data quality control

The data extraction tool was pre-tested on 5% of the sample size in other similar hospital with the assumption of similarity in documentation in the hospital settings. Data collectors were trained prior to data collection on the study objectives, eligibility criteria, reviewing registration logbooks and patient charts, practicing Kobo Tool Collect, and maintaining data confidentiality. The data was checked for missing information and completeness on a daily and weekly basis by the principal investigator and supervisor.

### Study variables

Independent variables: socio-demographic (age, sex, and residence), comorbid/antecedent risk factors (DM, cardiac and HTN problems, presurgical eye disease, chronic eye disease), behavioral factors (substance use), and clinical/surgical factors (level of visual acuity, type of cataract, eyes to be operated, types of lenses, post-operative complication, type of anaesthesia used for surgery, local anaesthesia, general anaesthesia, time taken for cataract surgery, types of cataract surgery).

The dependent variable: time to recovery from cataract surgery, was measured by the degree of visual acuity improvement and patient satisfaction with their new vision following cataract extraction with lens replacement or intraocular lens implantation [16].

### Operational definitions

Cataract recovery is the measurable restoration of visual acuity and patient satisfaction following cataract surgery, as determined by improvements in vision clarity and individual contentment with their post-operative eyesight. It was assessed through Visual Acuity Testing, Gathering feedback on visual symptoms, satisfaction with vision, and quality of life changes following the cataract surgery. It is measured by the degree of visual acuity improvement and patient satisfaction with their new vision following cataract extraction with lens replacement or intraocular lens implantation [15].

Event: recovery from cataract during the study period or regaining a normal state from cataract surgery within 1 year [16].

Censored: A cataract patient undergoing cataract surgery, but whose outcome is not observed within the designated follow-up period, or who is lost to follow-up or transferred to another healthcare facility

Loss to follow up: a patient who visit ophthalmic OPD three times and was appointed for the next visit but has not arrived on the date and his outcome was not recorded.

Time to recovery is defined as the time taken for a patient to achieve acceptable visual acuity [17].

Visual acuity: the ability to resolve detail at 6 m on a Snellen chart [18].

Blindness: a presenting visual acuity of <3/60 in the better eye [10].

Positive ocular history: if the participant had been diagnosed with eye disease of glaucoma, myopia, or had a previous history of ocular trauma [13].

Co-morbidity: any underlying health condition or disease that the patient had before the surgery in addition to cataract which includes DM, Hypertension, Chronic respiratory problems, and CVD [19].

Follow-up time: -From the time of admission until either an event or censorship occurs

Survival time:—is the measure of the follow-up time (in weeks) from the date of surgery up to the date of recovery, censored, or the end of the study

Post-operative complication: -was assessed by ‘Yes’ or ‘No’ questions, which was considered present if the cataract patients had complications like Bleeding, Slum/blurred vision, infection, inflammation, retinal detachment, swelling of the cornea, secondary cataract, glaucoma, dislocation of IOL [19].

Substance abuse: is the excessive, compulsive use of a substance, such as alcohol or drugs, leading to significant impairment in daily functioning, work, school, or relationships. This can be assessed through behaviors like frequent substance consumption, unsuccessful efforts to cut down or control use, and continued use despite negative consequences

#### Data processing and analysis

Data were collected or entered into Kobo Collect 4.0 and cleaned by using SPSS.25 and analyzed using STATA 16. Descriptive analysis was used to summarize the data. Bivariable logistic regression analysis was done to identify the statistical association between each independent and dependent variable. Kaplan–Meier curve was used to estimate median survival time, and cumulative probability of survival, and compare survival differences between the different covariates. The log-rank test was also used to compare statistical survival differences between categories of different explanatory variables. The variance inflation factor (VIF) helps assess how collinearity impacts the variance of regression coefficients. All independent variables have a maximum VIF of 6.7 indicates the absence of multicollinearity among the variables. The proportional hazard assumption was assessed using Kaplan Meier survival and Schoenfeld residual global test and the PH assumption was met (chi2 = 17.78 Prob>chi2 = 0.1226). Variables with a P-value<0.25 were entered into the multivariable Cox regression model analysis. Adjusted hazard ratio with 95% CI was calculated to determine the presence and strength of association among predictors and outcome variables. Finally, we consider the p-values less than 0.05 as statistically significant. Cataract patients who were transferred to other hospitals not developed outcomes in a study period were considered censored. However, the total time they contributed to the study was incorporated during the analysis of results.

### Ethics approval and consent to participate

This study was carried out in accordance with the Declaration of Helsinki relevant guidelines and regulations. The ethical committee which approved informed consent waiver was Hawassa university College of Medicine and Health Sciences Institutional Review Board with a letter reference number IRB/227/22, which currently chaired by Dr. Embialle Mengistie, and as members Dr. Achamyelesh Gebretsadik and Dr. Wondwosen Teklesilase (email address nembialle@hu.edu.et, agtsadik@yahoo.com, wondeti@yahoo.com) respectively. Hawassa University College of Medicine and Health Sciences Institutional Review Board members namely (Dr. Embialle Mengistie, Dr. Achamyelesh Gebretsadik and Dr. Wondwosen Teklesilase) were approved all protocols conducted in this study. Then, the written permission letter was also obtained from all the health administrative with reference number HCSH/198/14. Then data was collected from cataract surgery patient’s registry. The verbal consent was obtained from hospital managers in order to use individual cards for data collection. The retrieved data kept strictly confidential and names of patients were not included and never disclosed to others without informed consent of hospital. Medical record number was recorded rather than the patient’s name.

## Results

### Socio-demographic characteristics and behavioral factors of the study participants

Out of the 444 selected reviewed patients’ medical charts, 416 (93.6%) of them were found to be complete. Among 416 participants, half of them 211 (50.7%) were males and about one-third (37.5%) of the study participants were above the age of 60 years old. The median (IQR) age of participants was 55 (44 to 70) years old. Regarding the place of residence, nearly half of the cataract patients were 218 (50.25%) rural dwellers. Of all participants, 257 (61.77%) had a history of substance use (Table 1).

**Table 1:** Socio-demographic characteristics and behavioral factors of study participants with cataract at two selected Public Hospitals in Hawassa, 2023. (n = 416).

Variable	Category	Frequency (%)	Recovery (%)	Person-weeks	Incidence density
Age in year	1–15	45(10.81)	18(40)	1675	.010
	16–45	82(19.71)	75(91.47)	2283.71	.032
	46–60	133(31.97)	124(93.24)	3177.28	.039
	>60	156(37.5)	101(64.74)	5341.85	.018
Sex	Male	211(50.72)	162(76.77)	6167.42	.026
	Female	205(49.28)	156(76.09)	6310.42	.024
Residence	Urban	207(49.75)	195(94.21)	5578	.034
	Rural	209(50.25)	123(58.85)	6899.85	.017
Substance use	Yes	257(61.77)	201(78.20)	7502	.026
	No	159(38.23)	117(73.58)	4975.85	.023

### Clinical and surgical characteristics of the study participants

One hundred eighty-three (43.99%) participants were diagnosed with low preoperative visual acuity, 225 (54.09%) were diagnosed as age-related cataracts and three-fourth (75.72%) of the cataract patient had gotten small incision cataract extraction (SICE) surgery,361(86.78%) were locally anesthetized with 274 (76.7%) of RBA type of anesthesia. Concerning time taken for surgery 248 (59.62%) finished within forty-five minutes, for two-thirds of them 366 (87.98) posterior chambers were incised and 210 (50.48) surgery done for their left eye. Over 27.88% of the participants developed post-operative complications (Table 2).

**Table 2:** Clinical and surgical characteristics of study participants with cataract at two selected Public Hospitals in Hawassa, 2023. (n = 416).

Variable	Category	Frequency (%)	Recovery (%)	Person-weeks	Incidence density
Level of visual acuity	Low/poor	183(43.99)	126(39.6)	6297.14	.020
	Medium/borderline	132(31.73)	110(34.6)	3320.85	.033
	High/severe	101(24.27)	82(25.8)	2859.85	.028
Type of cataract	Age related	218(52.41)	158(72.47)	7078.14	.022
	Secondary	75(18.03)	67(89.34)	1881.42	.035
	Congenital	22(5.29)	9(40.90)	625.14	.014
	Traumatic	77(18.51)	70(90.90)	1995.14	.035
	Developmental	24(5.76)	14(58.34)	898	.015
Eyes to be operated	OD	180(43.27)	135(75)	5515.71	.024
	OS	210(50.48)	167(79.52)	6067.28	.027
	Both	26(6.25)	16(61.53)	894.85	.017
Type of lenses	PC	366(87.98)	279(76.22)	11053.28	.025
	Sulcus	34(8.17)	28(82.35)	1003.71	.027
	AC	16(3.85)	11(68.75)	420.85	.026
Post-operative complications	Yes	116(27.88)	33(28.44)	4814.85	.006
	No	300(72.12)	285(95)	7663	.037
Type of anaesthesia used for surgery	LA	361(86.78)	281(77.83)	10596.28	.026
	GA	55(13.22)	37(67.27)	1881.57	.019
Local anaesthesia	RBA	275(76.17)	214(77.81)	7809.42	.027
	PBA	83(22.99)	61(73.49)	2670.28	.023
	Sub tenon	3(0.84)	1(33.34)	62.71	.031
General anaesthesia	Sedative	53(96.37)	36(67.92)	1877.28	.019
	LMI	2(3.63)	1(50)	4.28	.233
Time taken for cataract surgery	1–45	248(59.62)	190(76.61)	7126.28	.026
	> 45	168(40.38)	128(76.19)	5351.57	.023
Type of cataract surgery	LWO	101(24.28)	75(74.25)	3019	.024
	SICS	315(75.72)	243(77.14)	9458.85	.025

**Key:—OD** = Oculus Dexter (right eye) **OS** = "Oculus Sinister"(left eye) **LWO** = Lens washout **PC** = Posterior Chamber **AC** = Anterior Chamber **LA** = Local Anesthesia **GA** = General Anesthesia **RBA** = Retro Bulbar Anesthesia **PBA** = Peri Bulbar Anesthesia **IOL** = Intraocular Lens (IOL) **SICS** = Small Incision Cataract Surgery **LMA** = Laryngeal Mask Insertion anesthesia

### Comorbid medical diseases before or after surgery of the study participants

**Fig 1:**
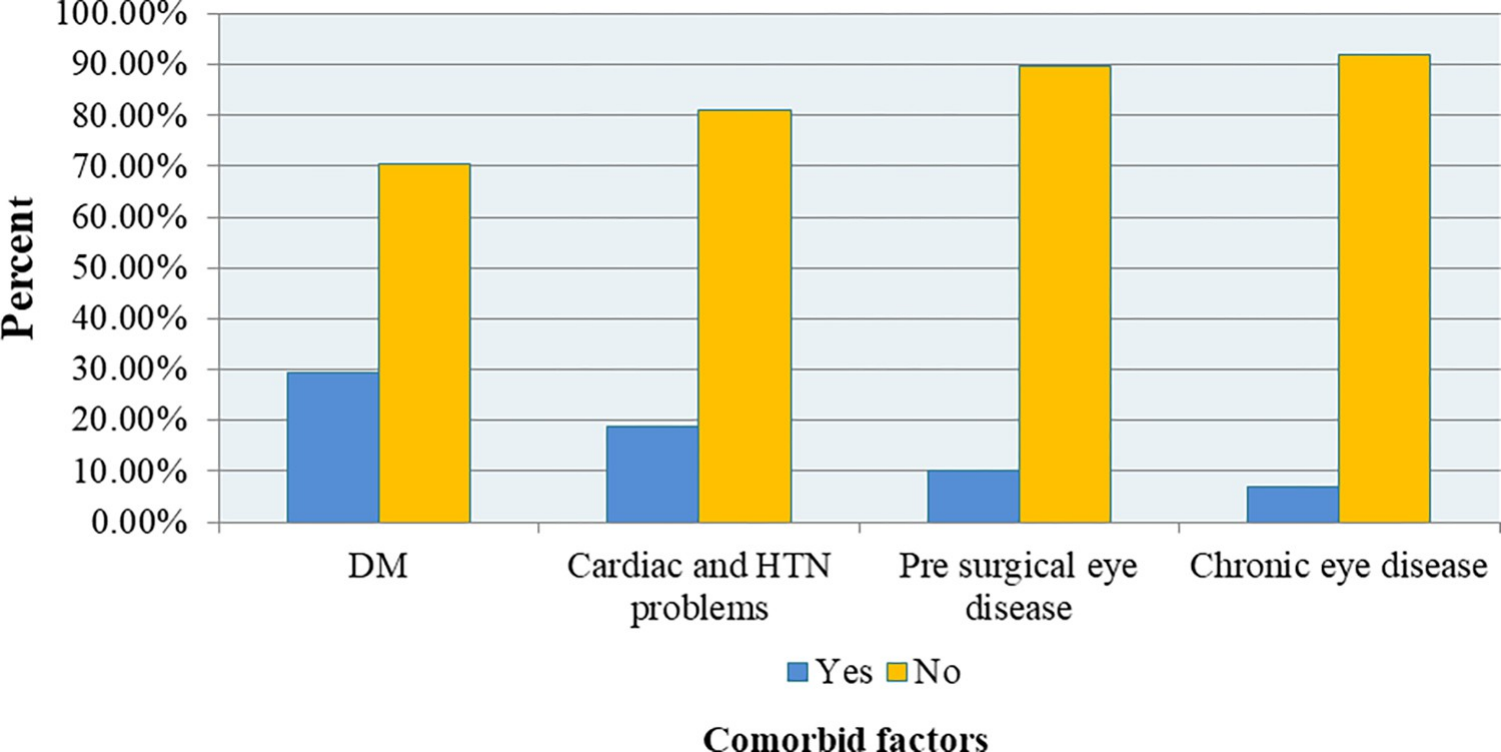
Comorbid medical diseases before or after cataract surgery of the study participants admitted at two selected Public Hospitals in Hawassa, Sidama Ethiopia, 2023. (n = 416).

Among the 416 participants, nearly one-third of them 29.4% had diabetes mellitus, near to two-tenth of them 18.9% had a history of cardiac/hypertension problems, 10.28% had previous other eye disease, and 6.93% with chronic eye disease (Fig 1).

### Incidence and Kaplan Meir survival estimates for cataract surgery recovery

During follow-up, among the patients treated with cataract surgery 318 (76.14%) recovered from cataract, with 95% CI (72–80). The total person-time followed was 12,477.85 person-weeks. The overall rate of recovery was 2.5 per 100 person weeks (95% CI: 2.2–2.8).

The overall median (IQR) survival time was 30 weeks with (95% CI, 26.28–33.85). The shortest and the longest time to recover from cataracts were 3 and 54 weeks respectively.

**Fig 2:**
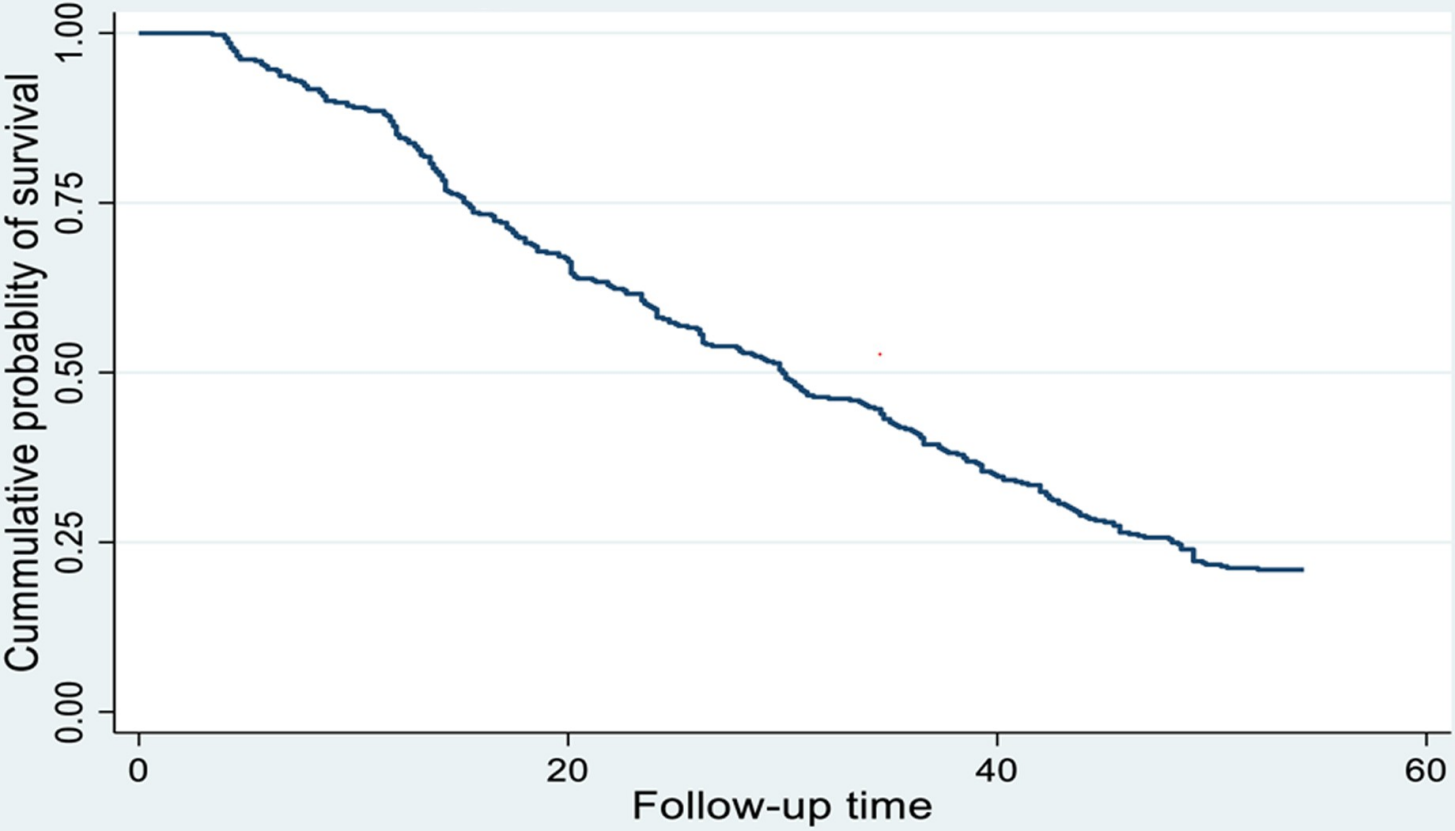
Overall Kaplan-Meier survival estimate of recovery time among cataract patients treated with cataract surgery at two selected Public Hospitals in Hawassa, Ethiopia, 2023. (n = 416).

**Fig 3:**
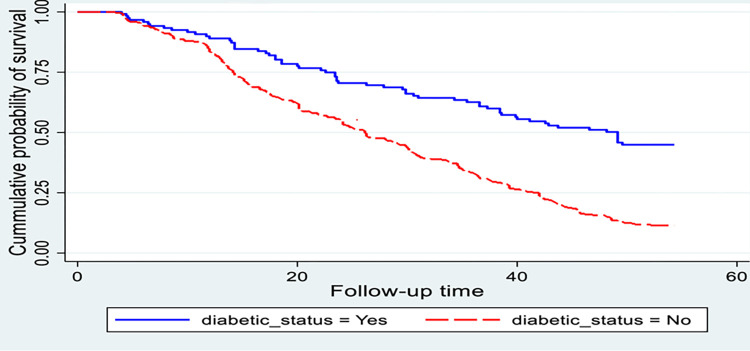
Kaplan-Meier survival estimate for time to recovery among cataract patients treated with cataract surgery, sub grouped by diabetic mellitus status. (n = 416).

**Fig 4:**
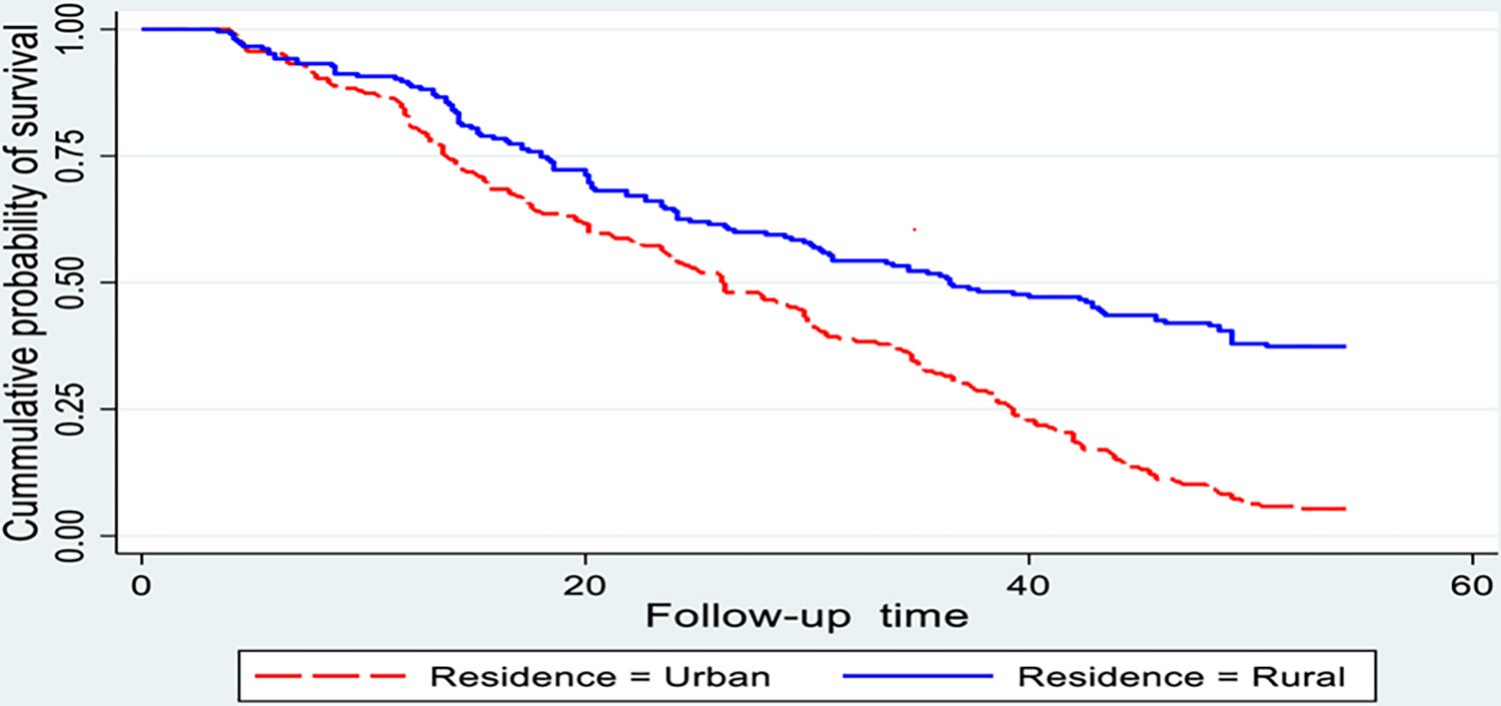
Kaplan-Meier survival estimate for time to recovery among cataract patients treated with cataract surgery, sub grouped by residence. (n = 416).

**Fig 5:**
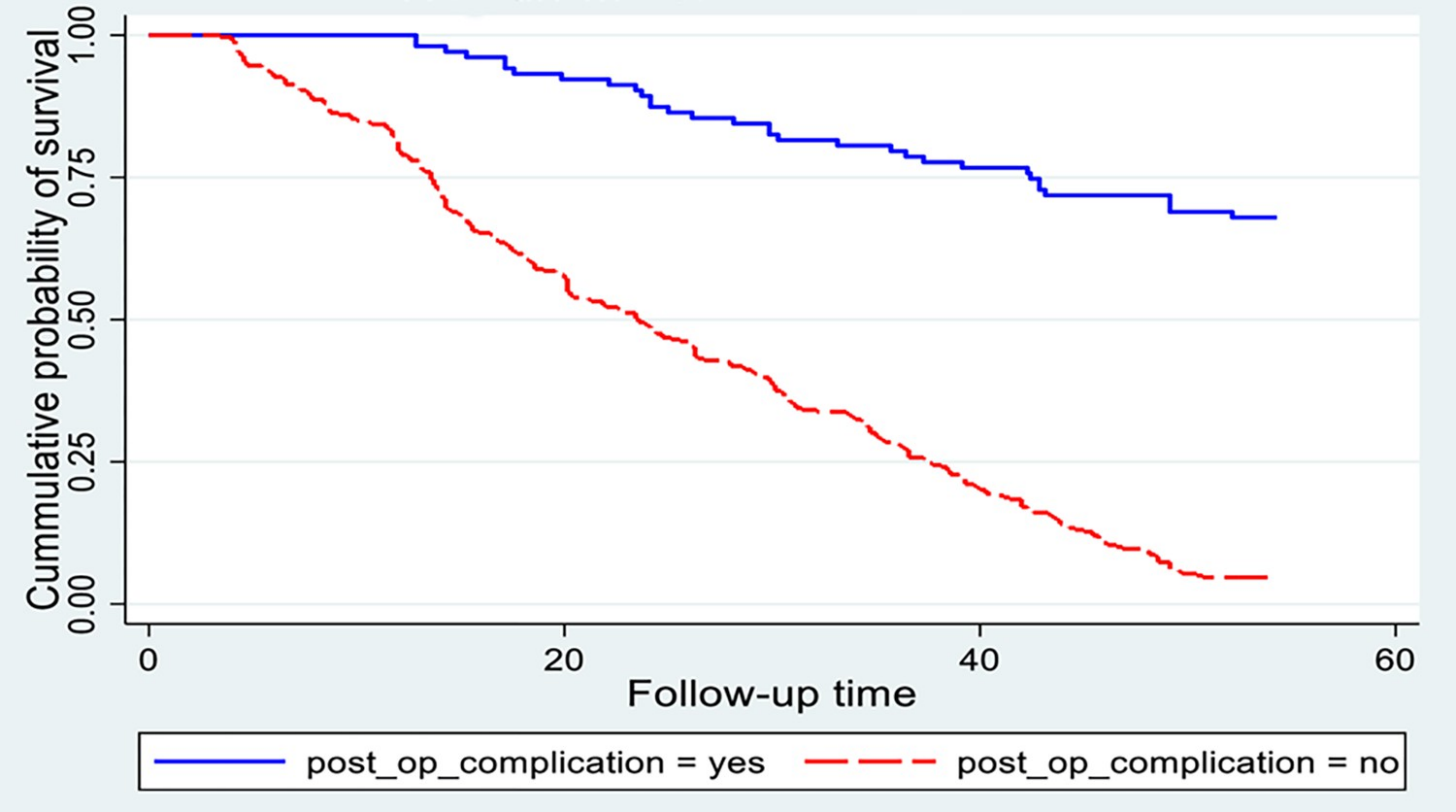
Kaplan-Meier survival estimate for time to recovery among cataract patients treated with cataract surgery, sub grouped by post-operative complication. (n = 416).

**Fig 6:**
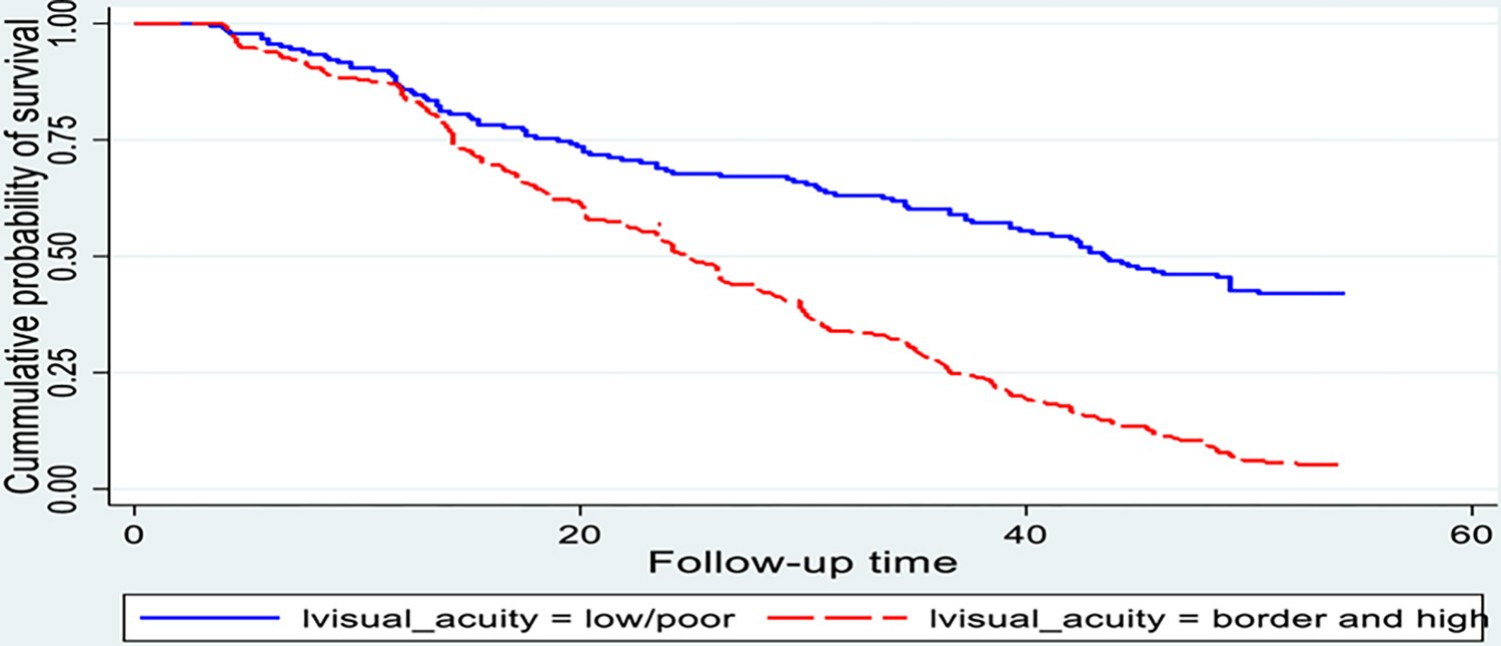
Kaplan-Meier survival estimate for time to recovery among cataract patients treated with cataract surgery, sub grouped by presurgical visual acuity. (n = 416).

The Kaplan-Meier survival curve was used to estimate the survival status of cataract patients within the first year; the curve tends to decline somehow slowly, implying that most cataract patients have not recovered from their illness within this time frame (Fig 2). Cataract patients’ survival estimates varied depending on their DM status, residence, and presurgical complication (Figs 3–6).

A significant recovery time difference has been observed between cataract patients, who had diabetes mellitus were 49 weeks compared to their counterparts at 26 weeks This difference was statistically significant with a p-value < 0.001 (Fig 3).

Similarly, there was a significant difference in recovery time between rural dwellers and their counterparts 36 weeks and 26 weeks respectively. This difference was statistically significant with a p-value < 0.001 (Fig 4).

There was a significant difference between those patients who had a post-operative complication, and were unable to recover within the time frame and those who didn’t have post-operative complications during or after surgery takes 23 weeks. This difference was statistically significant with a p-value < 0.001 (Fig 5).

Similarly, there was a significant difference in recovery time between patients who had a low presurgical visual acuity at 43 weeks and their counterparts at 24 weeks. This difference was statistically significant with a p-value < 0.001 (Fig 6).

### Predictors of recovery time from cataract

In the Bivariable Cox regression analysis age, residence, diabetic mellitus, substance use, level of visual acuity, chronic respiratory problems, type of anesthesia, type of cataract, surgery time, and post-operative complication were used to estimate their crude hazards ratio (CHR).

In multivariable Cox proportional hazard regression age, residence, diabetic mellitus status, presurgical visual acuity, type of cataract, and post-operative complication were estimated as independent predictors of time to recovery.

In a study conducted on cataract patients, several factors were found to be associated with the duration of survival after surgery. The results revealed that patients between the ages of 46 and 60 years had a 2.04 times higher likelihood (AHR = 2.04, 95% CI = 1.52–2.66, P = 0.001) of surviving for a shorter duration compared to those above 60 years of age.

Furthermore, patients residing in urban areas were found to be 1.48 times more likely (AHR = 1.48, 95% CI = 1.13–1.93, P = 0.004) to experience a shorter survival duration when compared to patients living in rural areas. Additionally, cataract patients who presented with borderline/high visual acuity had a 1.49 times higher likelihood (AHR = 1.49, 95% CI = 1.13–1.93, P = 0.003) of surviving for a shorter duration than those with poor visual acuity at presentation.

Moreover, patients presenting with secondary cataract or traumatic cataract were found to have 1.56 times (AHR = 1.56, 95% CI = 1.11–2.18, P = 0.009) and 1.82 times (AHR = 1.82, 95% CI = 1.32–2.52, P = 0.001) higher likelihood, respectively, of experiencing a shorter survival duration compared to patients with age-related cataract.

Interestingly, patients with diabetes mellitus exhibited a 32% lower pace of recovery (AHR = 0.68, 95% CI = 0.50–0.92, P = 0.012) when compared to those without diabetes mellitus. Lastly, patients who experienced postoperative complications faced a significant delay in recovery, with a 77% increased duration (AHR = 0.23, 95% CI = 0.15–0.35, P = 0.001) compared to those without any postoperative complications (Table 3).

**Table 3:** Predictors of time to recovery among cataract patients with cataract at two selected Public Hospitals in Hawassa, 2023. (n = 416).

Variable	Category	Recovery (%)	Follow-up time (person-weeks)	CHR 95% CI	AHR 95% CI
Age in year	1–15	18(40)	1675	0.54(0.33–0.90)	0.54(0.27–1.08)
	16–45	75(91.47)	2283.71	3.47(2.07–5.81)	1.13(0.78–1.65)
	46–60	124(93.24)	3177.28	4.51(2.73–7.43) **	**2.01(1.52–2.66)** **
	>60	101(64.74)	5341.85	1	1
Residence	Urban	195(88.4)	5578	2.21(1.75–2.70) **	**1.47(1.12–1.92)** *
	Rural	123(64.4)	6899.85	1	1
Substance use	Yes	201(78.20)	7502	1	1
	No	117(73.58)	4975.85	0.18(0.92–1.46)	0.95(0.72–1.26)
DM status	Yes	63(51.22)	4411	0.40(0.30–0.52) **	**0.68(0.50–0.92)** *
	No	255(87.03)	8066.85	1	1
Level of visual acuity	Low	100(54.64)	6408.42	1	1
	Medium /High	218(93.56)	6069.42	2.73(2.14–3.49)	**1.49(1.14–1.94)** *
Chronic respiratory disease	Yes	18(62.06)	920.85	1.35(0.84–2.18)	0.99(0.60–1.64)
	No	300(77.51)	11557	1	1
Type of anaesthesia	LA	281(77.83)	10596.28	1	1
	GA	37(67.27)	1881.57	1.41(1.00–1.99) *	0.77(0.49–1.22)
Post-operative complications	Yes	33(28.44)	4814.85	0.13(0.09–0.19) **	**0.23(0.15–0.35)** **
	No	285(95)	7663	1	1
Time taken for cataract surgery	1–45	190(76.61)	7126.28	1	
	> 45	128(76.19)	5351.57	0.86(0.68–1.07)	0.23(0.72–1.16)
Type of cataract	Age related	158(72.47)	7078.14	1	1
	Secondary	67(89.34)	1881.42	1.74(1.31–2.33) **	**1.56(1.11–2.18)** *
	Congenital	9(40.90)	625.14	0.63(0.32–1.24) *	1.70(0.80–3.63)
	Traumatic	70(90.90)	1995.14	1.68(1.26–2.23) **	**1.82(1.32–2.52)** **
	Developmental	14(58.34)	898	0.67(0.39–1.16)	1.69(0.88–3.23)

Note:

* variables significant with p-value ≤0.05

** shows significant difference at p<0.001, 1 = reference category; **CHR** = crude hazard ratio; **AHR** = adjusted hazard ratio; **CI** = confidence interval, **LA** = Local Anesthesia **GA** = General Anesthesia

## Discussion

This study was conducted to determine the time to recovery from cataract surgery and its predictors. The overall median time to recovery was 30 weeks. Age of the patients, residence, diabetes mellitus, initial level of visual acuity, types of cataracts, and postoperative complications were independent predictors of time to recovery.

The average duration of recovery from cataracts was found to be 30 weeks in our study, consistent with research from Ethiopia (23 weeks) [9]. In contrast, studies from Eastern Finland (17 weeks) [8] and Sun Yat-sen University in China (16 weeks) [20] reported shorter recovery times. This disparity in findings may be attributed to various factors such as differences in healthcare practices, patient demographics, or treatment protocols [21].

Additionally, variations in follow-up duration and method of assessing recovery could also contribute to the observed difference between the studies, and it’s worth noting that the term recovery itself may have different interpretations across studies [7]. Some studies may define recovery as the time taken to achieve improved visual acuity [17], while others may consider it as the duration required for patients to resume normal daily activities without significant visual impairment [8].

Adult cataract patients aged from 46 to 60 years had faster recovery as compared with older individuals. The finding is supported by a study conducted in India [22] and China [23]. The possible reason in this study could be due to age-related differences in healing capacity and physiological responses playing a role in recovery time [24]. Additionally, it could be due to an older age, individual having a chance of coexisting ocular pathology and having associated medical co-morbidity and may have a higher prevalence of mild systemic conditions. These factors could be potentially delaying the recovery process following cataract surgery [25].

The recovery times among cataract patients who live in urban had 48% faster recovery as compared with rural dwellers. This finding is supported by other studies conducted in the South Indian state of Andhra Pradesh [26] and a similar study in Ethiopia [9], implying that cataract patients who live in urban fasten recovery compared with rural dwellers. This could be because the patients in urban areas had better access to healthcare facilities and specialized care, which may end up with faster visual recovery following cataract surgery [27] and patients who live in rural areas may have limited access to specialized ophthalmic care, longer travel to a health care facility and potential delays in postoperative follow-up appointments [28].

Cataract types such as secondary cataracts and traumatic cataracts had faster recovery time as compared to age-related cataracts. This finding is supported by other studies conducted in India [29] and a similar study conducted in Ethiopia [9]. These findings suggest that different cataract types may have varying effects on recovery time, likely due to differences in the underlying causes and associated surgical considerations [26]. Additionally, the possible reason might be age-related cataracts associated with coexisting ocular pathology causing loss of vision and age causes degeneration of lenses [19].

Regarding visual acuity, Cataract patients with Borderline/ Good presurgical visual acuity had 49% faster recovery as compared with those who had poor presurgical visual acuity. This finding was supported by another study done in China [28], and Ethiopia [9]. The possible reason might be that patients with better presurgical visual acuity may have a more intact optical system, including the optic nerve and visual pathways this can facilitate faster neural adaptation and adjustment to the new artificial lens after cataract surgery, resulting in a quicker recovery of visual function [30].

Cataract patients with diabetes mellitus had a 32% lower pace of recovery when compared with those without diabetes mellitus. A similar finding of the study was supported by the study done in Finland [8] and Ethiopia [22]. This could be because diabetes can affect the body’s ability to heal wounds and recover from surgical procedures and it can impair the healing process by reducing blood flow and impairing immune function [8].

Cataract patients who had postoperative complications had 77% of delay recovery as compared with those who had no post-operative complications. This finding is supported by a study done in Eastern Finland [8] and another randomized trial study [18]. The possible reason might be patients who face post-operative complications often require additional medical interventions such as medications, procedures or prolonged hospital stays these interventions may introduce additional factors that can delay the overall recovery process [31].

In contrast, other studies done in France [32] reported positive outcomes despite the presence of postoperative complications. The possible reason might be due to advances in surgical technique, technology, and pre-and post-operative care have significantly improved the overall outcomes of cataract surgery [30]. Additionally, prompt identification and management of complications can help to minimize their impact on the final visual outcome, and loss monitoring and appropriate treatment by the surgical team can address the issues as they arise [12].

## Strength and limitation the study

The strength of this study was that being a cohort study could have helped to measure the temporal relationship between the exposure variables and the time to recovery from cataract surgery. Despite of its being a retrospective study, we were unable to found some important variables such socioeconomic status and lifestyle habits that should be considered to identify predictors. Moreover, the visual recovery data for children might be biased as it could be taken from parents/care-givers. Hence, the findings were interpreted with caution.

## Conclusion

In the study area, the time to recovery was slightly high. Age of the patients, place of residence, initial visual acuity status, cataract types, and postoperative complications were the predictors of time to recovery following cataract surgery. These findings emphasize the need to consider these factors in patient evaluation and postoperative care for optimal recovery in cataract surgery. Policy makers and health care practitioners should focus towards preventing and managing comorbidities and complications alongside cataract surgery to enhance the recovery period.

## Supporting information

S1 Data (DOCX)

S1 Dataset (XLS)

## Abbreviations

AHR: Adjusted Hazard Ratio
BCVA: Best Corrected Visual Acuity
CHR: Crude Hazard Ratio
CI: Confidence Interval
CVD: Cardio Vascular Disease
CSR: Cataract Surgical Rate
CSC: Cataract Surgical Coverage
ECCS: Extra Capsular Cataract Surgery
ECCE: Extracapsular cataract extraction
ICCE: Intracapsular cataract extraction
IOL: Intraocular lens
IRB: Institutional Review Board
SPSS: Statistical Package for Social Science
SICS: Small-Incision Extracapsular Cataract Surgery
WHO: World health organization
WFSA: World Federation of Societies of Anesthesiologists
