# Supplementary material for: Time to recovery following cataract surgery and its predictors among patients undergoing surgery at two selected Public Hospitals in Hawassa, Sidama, Ethiopia
Source: PLoS One. 2024 Nov 4;19(11):e0313118. doi: 10.1371/journal.pone.0313118 (PMC11534261; doi:10.1371/journal.pone.0313118)
Supplement: S1 Data — (DOCX) [file pone.0313118.s001.docx]

**Data extraction checklist**

**Title: “*Time to Recovery from Cataract Surgery and Its Risk Factors among Patients Treated with Surgery in Hawassa Public Hospitals, Sidama, Ethiopia”2023*.**

This tool is developed for the collection of data that are essential for the assessment of recovery time and its predictors among patients with Cataract admitted to selected public hospitals in Hawassa, Ethiopia, 2022. All relevant information to the study will be retrieved from the client’s chart without stating their name. Health care professionals (BSc Nurses) working in the ophthalmic ward will collect this data and will be kept confidential.

**Part-I Socio Demographic Characteristics**

| **No** | **Variables** | **Possible answers** | **Skip** |
| --- | --- | --- | --- |
| 101 | Age | ______years or  ______months |  |
| 102 | Sex | 1. Male 2. Female |  |
| 103 | Residence | 1. Urban 2. Rural |  |

**Part-II Comorbid medical factors**

| **No** | **Variables** | **Possible answers** | **Skip** |
| --- | --- | --- | --- |
| 201 | Diabetes mellitus | 1.Yes 2 No |  |
| 202 | Cardiac problems | 1.Yes 2 No |  |
| 203 | Hypertension | 1.Yes 2 No |  |
| 204 | History of other eye disease | 1.Yes 2 No |  |
| 205 | Level of visual acuity | 1. Low 2. Medium 3. High |  |

**Part-III Behavioral factors**

| **No** | **Variables** | **Possible answers** | **Skip** |
| --- | --- | --- | --- |
| 301 | Smoking status | 1.Yes 2 No |  |
| 302 | Alcohol consumption | 1.Yes 2 No |  |

**Part-IV Clinical/surgical factors**

| **No** | **Variables** | **Possible answers** | **Skip** |
| --- | --- | --- | --- |
| 401 | Type of cataract identified |  |  |
| 402 | Type of cataract surgery |  |  |
| 403 | Type of anaesthesia done | 1. Local anaesthesia 2. General anaesthesia |  |
|  | If local | 1. RBA 2. PBA 3. SUBTENON |  |
|  | If general | 1. Sedation 2. LMI |  |
| 404 | Type site lens inserted | 1. PC 2. SULCUS 3. AC |  |
|  | Date of surgery | ________/_________/______(DD/MM/YY) |  |
|  | Date of recovery | ________/_________/______(DD/MM/YY) |  |
|  | Outcome status | Recovered Transferred out  Not recovered Loss follow up |  |
